# Distress and disability in young adults presenting to clinical services with mood disorders

**DOI:** 10.1186/2194-7511-1-23

**Published:** 2013-10-17

**Authors:** Elizabeth M Scott, Daniel F Hermens, Sharon L Naismith, Adam J Guastella, Django White, Bradley G Whitwell, Jim Lagopoulos, Jan Scott, Ian B Hickie

**Affiliations:** Clinical Research Unit, Brain & Mind Research Institute, The University of Sydney, 100 Mallett Street, Camperdown, New South Wales 2050 Australia; School of Medicine, The University of Notre Dame, 160 Oxford Street, Darlinghurst, Sydney, New South Wales 2010 Australia; Academic Psychiatry, Institute of Neuroscience, Newcastle University, Newcastle upon Tyne, NE1 7RU UK; Centre for Affective Disorders, Institute of Psychiatry, De Crespigny Park, London, SE5 8AF UK

**Keywords:** Bipolar, Unipolar, Youth, Alcohol and substance misuse, Co-morbidity

## Abstract

**Background:**

Distress and/or dysfunction are well established as key reasons for help-seeking. We explore the characteristics of groups defined by high or low distress or disability in young people with unipolar depression (UP) or bipolar disorder (BD).

**Methods:**

Individuals aged 12 to 25 years presenting to youth mental health services for the first time with a primary diagnosis of UP or BD were assessed using the Kessler Psychological Distress Scale (Kessler-10) and the Work and Social Adjustment Scale (WSAS). Four groups with high or low distress or impairment were defined (according to scores above or below the group medians for the Kessler-10 and WSAS). Multinomial logistic regression (MNLR) was used to examine how cases with high levels of distress and disability (reference group) differed from the other three groups.

**Results and discussion:**

The sample comprised 1,746 cases (90% UP, 56% female) with a median age of 17.5 years. Median scores on the Kessler-10 and WSAS were both high (30 and 20, respectively) and were significantly inter-correlated (*r* = 0.62); the high impairment/distress group was the largest sub-group (39% of cases). The MNLR analysis demonstrated that younger age was associated with lower impairment groups (irrespective of distress level), whilst male gender was associated with lower distress (irrespective of impairment). Compared to the low impairment/distress cases, the high impairment/distress group was significantly more likely to use cannabis and/or alcohol. Age, substance use and possibly gender are probably better predictors of distress/impairment sub-group than mood disorder sub-type in youth.

## Background

In recent decades, there has been a steady increase in the provision of early intervention services for young adults at risk of or in the early stages of psychosis. This has shed light on the reasons why some individuals with psychotic, mood or other mental disorders seek help, whilst other young people do not. For example, youth with psychosis or psychotic-like experiences are more likely to access clinical services if they perceive a greater need for care (because of a decline in personal functioning) and/or because they wish to ameliorate the distress associated with acute symptoms (Stip and Letourneau 2009; Yung et al. 2006). These clinical studies indicate that the reasons for presentation are dictated more by distress and dysfunction than by diagnosis alone. Further support for this hypothesis derives from a large-scale community health survey of more than 36,000 people aged ≥15 years that identified that seeking treatment was prompted by increasing levels of disability, distress and/or suicidal ideation and that these factors operated across diagnostic and socio-demographic groups (Sareen et al. 2005).

There are fewer studies of the clinical profile of youth presenting to care services with a mood disorder, but data on middle-aged adults with unipolar depression (UP) and bipolar disorder (BD) suggest that distress is a robust predictor of help-seeking (Angst et al. 2010; Mojtabai et al. 2002). Impairment also increases the likelihood of presentation to clinical services by older people, especially in those with co-existing substance misuse or sub-threshold syndromes (Elhai and Ford 2007; Murphy et al. 2010). However, those studies do not enable us to understand whether young people presenting with BD demonstrate similar patterns of distress and/or impairment to those reported by individuals with UP. Further, it is not clear if disability in youth is independent of or directly correlated with distress, nor do we have evidence about the characteristics of individuals with high levels of distress but low levels of impairment or vice versa.

A major barrier to understanding the issues outlined has been the lack of access to samples of adequate size or that are truly representative of youth who are seeking help for unipolar or bipolar mood or other mental disorders at the time of first clinical presentation. In 2006, the Australian Government launched '*headspace*’ services in order to extend access to mental health care for young people aged 12 to 25 years (McGorry et al. 2007). Besides the clinical and social benefits of this initiative, there are also opportunities for research on emerging mood problems. In Sydney, preliminary studies of young people presenting to two *headspace* services highlighted that social impairments exist in the early, sub-syndromal stages of mood disorders (Hamilton et al. 2011) and that young people with psychotic, mood and/or anxiety disorders present with significant levels of distress (Scott et al. 2012). Also, a recent multi-site study (sample >2,000) identified that young people aged 12 to 30 years reported high lifetime rates of alcohol (76%), nicotine (62%) and cannabis use (54%), with the age at first consumption (mean ~ 15 years) preceding clinical presentation with a specific mental health problem by about 4 years (Hermens et al. 2013).

This study examined the levels of distress and impairment in over 1,600 individuals presenting to youth mental health services with a primary diagnosis of UP or BD and explored which combination of demographic, clinical and substance use parameters identified cases with high or low levels of distress and/or high or low levels of functioning.

## Methods

The Human Research Ethics Committee (University of Sydney) approved this study, and all patients gave prospective written informed consent for their clinical data to be used for research purposes. Parental consent was obtained for patients under 16 years of age. Patients who did not wish to participate were not required to explain withholding consent.

### Participants

Consecutive consenting cases aged 12 to 25 years who presented at two youth-specific mental health clinics with a probable primary diagnosis of UP or BD were recruited to the study between October 2007 and January 2012. Exclusion criteria were as follows: medical instability (as determined by a psychiatrist), lack of capacity to give informed consent, history of neurological disease (e.g. tumour, head trauma, epilepsy), medical illness known to impact cognitive and brain function (e.g. cancer) and/or clinically assessed IQ below 70 and/or insufficient English to participate in the research protocol.

### Assessments

Each referral participated in the interview-based intake procedure. As described previously (for full details, see Hamilton et al. 2011; Scott et al. 2012), as well as the standard clinical assessments, the assessing clinicians also completed a structured *pro forma*. This information was used to confirm the clinical diagnosis; any cases where diagnosis was uncertain were discussed in a consensus meeting that included senior experts in early onset mood disorders (IH and ES); any diagnoses recorded were also regularly checked for accuracy by a senior researcher (i.e. by DH), furthermore. For this study, the following data were used:

1. Socio-demographics: age at presentation, gender, vocational status (defined as being in employment, education or training: full time, part time or vocationally inactive)
2. Diagnostic and Statistical Manual, 4th Edition (DSM IV) mood disorder diagnosis (UP or BD sub-type) and any lifetime co-morbidities meeting DSM IV criteria (defined as none, any anxiety disorder, any behavioural or developmental disorder, any other co-morbidity) (American Psychiatric Association 2000). In addition, the presence or absence of psychotic symptoms was noted (as a separate item). As suicidal ideation may be a significant contributor to distress, this was assessed on a 0 to 3 scale using the format employed in depression rating scales such as the Hamilton (no ideation to suicidal acts)
3. Alcohol and substance use: the frequency of use and misuse (of alcohol, nicotine, cannabis, amphetamines, etc.) for the previous 3 months was assessed from the data recorded on the Alcohol, Smoking and Substance Involvement Screening Test (ASSIST; Humeniuk et al. 2008). Data on age at first consumption (if available) and use of or abstinence from cannabis and/or nicotine and/or alcohol were examined in this study
4. Kessler-10 Psychological Distress Questionnaire (Kessler and Mroczek 1992; Kessler et al. 2002; Andrews and Slade 2001): This well-validated 10-item questionnaire has been widely used in community and clinical settings and has been applied to a range of diagnoses and clinical presentations in adolescent and adult populations. Each Kessler-10 question refers to the preceding month and is rated on a 1 to 5 scale (from none of the time to all of the time), and the total score ranges from 10 to 50, with higher scores indicating higher levels of distress and scores >30 associated with severe mental disorders (only about 2% general population score >29). Its utility is that it measures distress associated with symptoms rather than focusing on the symptoms associated with one specific disorder
5. Work and Social Adjustment Scale (WSAS; Mundt et al. 2002): The WSAS is a 5-item self-rated questionnaire that asks the patient to rate key aspects of their current functioning (ability to work, home management, social leisure, private leisure and close relationships over the preceding 4 weeks). The scale has been used previously in those with more severe mental disorders (Mundt et al. 2002) and has been found to have good psychometric properties (Mundt et al. 2002; Mataix-Colsa et al. 2005). Each item is rated on a 0 to 8 scale (from 'not at all’ to 'severely affected’), and a total score of >20 is regarded as indicating high levels of impairment.

### Statistical analyses

All data were entered into a study database and analysed using SPSS version 19. Analysis of variance (ANOVA) with Dunnett’s *post hoc* tests was used to test for significant differences in mean age (across groups). Pearson product moment correlations were used to examine for significant associations between scores of distress and disability, with partial correlations testing the associations when controlling for age, gender and diagnostic sub-type. Differences for categorical measures between groups (e.g. gender, diagnosis, vocational status, ASSIST ratings) were assessed using chi-squared tests. Cases with missing data were excluded list-wise from the analyses. All statistical tests were two-tailed and used a significance level of *α* = 0.05.

Multinomial logistic regression (MNLR) was used to examine for demographic and clinical predictors of membership of four groups defined by a combination of high and/or low levels of impairment or distress (categories were defined by scores above or below the median for the Kessler-10 or WSAS). The largest group (which was the one with the highest level of impairment and distress) was selected as the reference category, and the forward (likelihood ratio) procedure was used for the regression. Variables were entered into the model if they had a significance level <0.05. The factors and variables included were as follows: age (split into categories according to 'terciles’), gender, mood disorder sub-type (BD vs UP), ASSIST ratings of the frequency of nicotine, cannabis and alcohol use (5-point rating: never, 1 to 2 occasions, monthly, weekly, daily) and the presence or absence of co-morbidities. Predictors of sub-group membership are reported using odds ratios (OR) with 95% confidence intervals (CI).

## Results

The sample comprised 1,746 individuals with 1,571 UP (90%) and 977 females (56%). At first presentation to clinical services, the sample mean age was 17.8 years (SD 3.4, median 17.5), with 33% of cases’ ages allocated to the following age groups: 12 to 15, 16 to 19 and 20 to 25 years, respectively. The median score on the Kessler-10 was 30, and on the WSAS, it was 20. Kessler-10 scores were statistically significantly correlated with WSAS scores (*r* = 0.62, *p* = 0.001), and this correlation remained statistically significant after controlling for age, gender and diagnostic sub-type (*r* = 0.49, *p* = 0.003). The sample median score for suicidal ideation was low (0.42, on a 0 to 3 rating), but 7% of cases (*n* = 124) expressed ideas of self-harm. Scores on the suicidal ideation scale and Kessler Psychological Distress Scale (K-10) were correlated (*r* = 0.51, *p* = 0.002), but there was no significant association with age, gender or diagnostic sub-type. About 5% of cases (*n* = 87) had experienced one or more psychotic symptoms, but again, there were no statistically significant associations with age, gender or diagnosis. Age at first consumption of alcohol, tobacco and cannabis was reported for only 962 of the sample, but the median for all substances was similar (about 14.5 years; 25th and 75th percentiles: 11 and 16 years, respectively), with females starting marginally but not significantly later than males (median: females, 15 years; males 14.5 years). There was no evidence of any differences in age at first substance use in sub-groups defined according to age at presentation (12 to 15, 16 to 19 and 20 to 25 years) although a larger proportion of the youngest age group had never consumed any of these substances.

**Figure 1:**
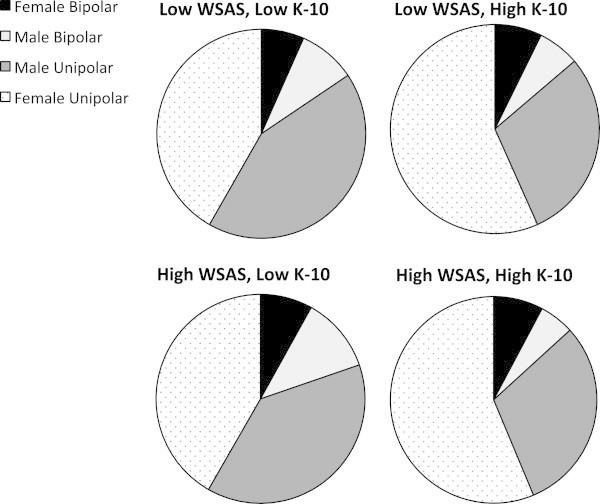
**Distribution of cases of unipolar and bipolar disorders in males and females across groups.** Defined by high and low scores on the WSAS and K-10.

Data was available on Kessler-10 and WSAS scores for 1,640 individuals (580 male UP, 124 male BD, 815 female UP and 121 female BD), allowing these cases to be allocated to one of the four distress/impairment sub-groups. Cases with high distress and high impairment (Kessler-10 score >30, WSAS score >20) formed the largest group comprising 39% of this sample, with just under a third of the sample classified in the high distress/low impairment (15%) or low distress/high impairment (14%) groups and the remainder (32%) categorised as having low distress/low impairment (see Table 1). As shown in Figure 1, the distribution of cases between the distress/impairment groups differed significantly by gender and diagnosis (*χ*^2^ = 31.29, *df* = 9, *p* = 0.001), with over 40% females (with UP or BD) allocated to the high distress groups (irrespective of impairment level) and about the same proportion of males (with UP or BD) classified as having low levels of distress (irrespective of impairment).

**Table 1:** **Basic characteristics of 1,640 cases allocated to groups according to levels of distress and impairment**

	Low impairment and low distress	Low impairment and high distress	High impairment and low distress	High impairment and high distress	Statistical test	Significance (***p***value)
	(***N*** = 527)	(***N*** = 247)	(***N*** = 223)	(***N*** = 643)		
Mean age (SD) in years (*n* = 1,678)^a^	17.07 (3.45)	17.40 (2.95)	18.31 (3.69)	18.53 (3.02)	*F* = 16.18	0.001
					*df* = 3, 1,674	
No vocational activity (*n* = 386)	81 (21%)	66 (17%)	54 (14%)	185 (48%)	*χ* ^2^ = 26.56	0.001
					*df* = 6^c^	
No lifetime co-morbidities (*n* = 512)	175 (33%)	75 (30%)	71 (33%)^b^	191 (29%)	*χ* ^2^ = 14.43	0.1
					*df* = 9^d^	
Nicotine abstinence (*n* = 894)	331 (63%)	134 (54%)	125 (58%)^b^	304 (47%)	*χ* ^2^ = 21.01	0.01
					*df* = 3	
Alcohol abstinence (*n* = 555)	216 (41%)	83 (34%)	89 (42%)^b^	167 (26%)	*χ* ^2^ = 24.93	0.01
					*df* = 3	
Cannabis abstinence (*n* = 1,177)	411 (78%)	177 (72%)	165 (77%)^b^	424 (66%)	*χ* ^2^ = 19.62	0.01
					*df* = 3	
Abstinence from substance use^b^ (*n* = 543)	217 (41%)	92 (37%)	87 (41%)^b^	147 (23%)	*χ* ^2^ = 38.27	0.001
					*df* = 9^d^	

^a^Total cases = 1,678 due to missing data; *n* = 1,640 were assigned to distress/impairment groups (the remaining *n* = 38 cases had missing K-10 or WSAS scores). Numbers included in each analysis are given in brackets; levels of distress and impairment were determined by K-10 and WSAS scores, respectively. *Post hoc* analysis shows that the high impairment/distress group is significantly older than both low impairment groups; the high impairment/low distress group is also significantly older than the two low impairment sub-groups (age of high distress groups do not differ significantly; *ditto* for the low impairment groups). ^b^Substance refers to alcohol, nicotine or cannabis. ^c^Three categories for vocational status. ^d^Four sub-groups for co-morbidity and for poly-substance use (see the 'Methods’ section for details).

Individuals in the high distress/impairment group were more likely to be older (*F* = 16.18; *df* = 3, 1,674; *p* = 0.001). Youth in this group were less likely to be in part-time or full-time education, training or employment (*χ*^2^ = 26.56, *df* = 6, *p* = 0.01), with almost 50% of the group reporting no vocational activity. However, vocational status was significantly influenced by age group, with nearly all cases aged 12 to 15 years being in full-time or part-time education and the majority of these cases were allocated to the groups with the lowest levels of impairment.

Co-morbid mental disorders (mainly anxiety or developmental/behavioural disorders) were diagnosed in over 65% cases (*n* = 1,187), but the prevalence and types of problems were similar across the four distress/impairment sub-groups. About a third of the sample (544/1,669) reported total abstinence from substance use (i.e. they had never used nicotine, alcohol or cannabis), and a significantly greater proportion of these cases (217/544; 40% of non-users) were allocated to the low distress/low impairment group. In contrast, users of any or all three substances (cannabis use = 29%, nicotine use = 47%, alcohol use = 67%, poly-substance use = 23%) were over-represented in the groups with high levels of distress, irrespective of their reported levels of impairment on the WSAS.

As shown in Table 2, MNLR indicated that individuals allocated to the high distress/low impairment group were likely to be younger (OR for 12 to 15 age group, 3.14; 95% CI, 1.67 to 6.06) than those in the high distress/impairment group. The variables that best differentiated individuals with low distress/high impairment from the high impairment/distress group were as follows: being more likely to be male (OR, 2.05; 95% CI, 1.29 to 3.22) and less likely to use nicotine, alcohol or cannabis (OR for poly-substance use, 0.61; 95% CI, 0.44 to 0.83). In contrast, the individuals in the low impairment/distress group were significantly younger (OR for 12 to 15 age group, 3.72; 95% CI, 2.18 to 6.34), more likely to be male (OR, 2.20; 95% CI, 1.21 to 3.83) and being less likely to report cannabis (OR, 0.27; 95% CI, 0.19 to 0.45) or alcohol use (OR, 0.37; 95% CI, 0.16 to 0.81). An additional analysis of this latter group (available from the authors) that examined any interactions between gender and diagnosis showed a greater probability that males with BD were especially likely to be categorised in the low distress/impairment group rather than in the high distress/impairment group (OR, 3.1; 95% CI, 2.01 to 9.74).

**Table 2:** **Multinomial logistic regression**

Differences from high distress and high impairment	Significance (***p***value)	OR	95% confidence intervals
High distress and low impairment			
Age 12 to 15 years	0.001	3.14	1.63 to 6.06
High impairment and low distress			
Male	0.01	2.05	1.29 to 3.22
Poly-substance use^a^	0.003	0.61	0.44 to 0.85
Low impairment and low distress			
Age 12 to 15 years	0.000	3.72	2.18 to 6.34
Male	0.001	2.20	1.21 to 3.83
Alcohol use	0.004	0.37	0.16 to 0.81
Cannabis use	0.004	0.27	0.19 to 0.45

Differences between the group with a high level of distress and impairment (reference group) compared to groups defined by other combinations of high or low levels of impairment and of distress are demonstrated. ^a^'Poly-substance use’ refers to consumption of nicotine, alcohol and cannabis (see text for details).

**Figure 2:**
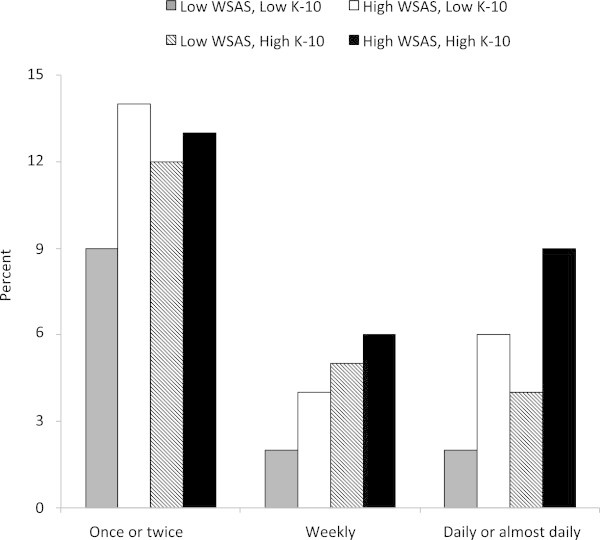
**Frequency of cannabis use in the last 3 months.** In groups defined according to high and low scores on the WSAS and K-10 scores.

Finally, we briefly examined the association between the rate of cannabis use by cases across the four distress/impairment sub-groups. Figure 2 shows the proportion of individuals in each group using cannabis at different frequencies (collapsed from five to three categories: less than monthly, less than weekly, daily or almost daily). The patterns of cannabis consumption suggest that whilst infrequent use (one to two occasions) does not differentiate between groups, consumption of cannabis on a weekly basis is associated with a 'dose-response’ profile, whilst the highest levels of consumption (daily or almost daily cannabis use) are associated with the groups with the highest levels of impairment, irrespective of distress level.

## Discussion

This study is one of the largest undertaken so far of a cohort of adolescents and young adults presenting for the first time to youth mental health services with a primary diagnosis of mood disorder. It is the first that tries to understand the characteristics of groups defined by levels of distress and disability at the time of presentation. Given the age range of the study population, it is unsurprising that 90% of cases are currently categorised as UP, as a third of the study sample have not yet entered the period of maximum risk for the onset of hypomanic or manic episodes (50% BD cases commence between 15 and 25 years), and most of the rest of the cases have not completed their transition through the peak age at the onset period. This is important to remember when considering the findings, especially with regard to the relative lack of differences in levels of impairment and/or distress between UP and BD. Further prospective follow-up is required to determine the trajectories of mood disorders in this cohort, to observe how many cases classified as UP later make a transition to BD and to identify if distress, impairment or other parameters have predictive validity.

Another important study finding is that young people with mood disorders presenting to youth mental health services do not have 'mild problems’. The mean and median scores of the distress (K-10) and functional ratings (WSAS) suggest that they have levels of distress and impairment that are comparable with those seen in youth with other severe mental disorders, and distress levels are similar to those reported in middle-aged and older adults (Angst et al. 2010; Elhai and Ford 2007; Mojtabai et al. 2002). Levels of co-morbidity are high, and some degree of substance use is reported by two thirds of the sample - which is again similar to that seen in adult populations with established mood disorders (Murphy et al. 2010). It was noteworthy that - as reported previously (Hermens et al. 2013) - the first use of alcohol or other substances preceded help-seeking for UP or BD by at least 2 years in the majority of cases. Younger age seems to be associated with marginally less functional impairment (measured on the WSAS) than older adult populations (Murphy et al. 2010), but many of those studies used different measures for impairment. However, even within our youth sample, the youngest age group (12 to 15 years) showed the lowest levels of impairment (irrespective of distress level). However, it is not possible to clarify if this is simply because they have been ill for a shorter period of time (compared to their older peers), because other people (e.g. teachers or parents) can be more influential in initiating referrals and/or these individuals are less likely to have a substance use habit. Clearly, these are important issues to disentangle in future studies.

Diagnostic sub-type alone did not differentiate groups defined according to high or low impairment or distress; indeed, it was noticeable (see Figure 1) that male BD cases were classified in the group with the lowest distress/impairment levels, whilst females with BD were more likely to be categorised as highly distressed/impaired. The cross-sectional nature of the study and the measures employed do not provide robust evidence as to whether females are sicker than males at first clinical presentation or that females delay help-seeking longer than males. The latter is certainly not in keeping with the published literature (Hickie et al. 2001; McGorry et al. 2007; Scott et al. 2012) and so other explanations need consideration. For example, the gender findings could be an artefact of sampling, as it is conceivable that females (even when highly distressed or impaired) are more likely to agree to participate in research than males. However, an alternative, more plausible interpretation is that females are more likely to acknowledge the degree of dysfunction caused by their symptoms or they perceive that their symptoms and disability lead to more severe levels of distress and impairment (thus scoring higher on the rating scales employed).

Lastly, the groups with the highest versus the lowest levels of distress/impairment were distinguished by the use of cannabis and alcohol. Furthermore, a brief review of Table 1 suggests that lower levels of distress (irrespective of impairment) were associated with abstinence from alcohol, cannabis, nicotine or poly-substance use. Along with the findings of the MLRN, it is indirect evidence that substance use aggravates the initial clinical presentation of a mood disorder. There was also preliminary evidence that the frequency of consumption (e.g. Figure 2 on the use of cannabis) was associated with a dose–response effect on distress and impairment. However, as there was limited data regarding the age at first use of this substance (many participants did not report this), it was decided that it was not appropriate to analyse the additional significance of duration of substance use nor the issue of primacy (did substance use precede mood disorders in the worst functioning/most distressed groups) or was it a secondary phenomenon, e.g. representing a maladaptive 'coping’ style. This study highlights the need for careful assessment of temporality of the onset of mood disorders and substance use and emphasises that any treatment strategies for youth presenting with UP or BD need to be designed with a view to their applicability to groups who have co-occurring substance use.

Finally, it is important to acknowledge the limitations to this study. For example, we did not assess neuropsychological functioning or medical illnesses, both of which may impair functioning or augment distress, nor did we assess personality disorders, although the value of assessment of these in younger adults is debatable, especially in those aged 12 to 15 years or so. It is also possible that young people with psychotic or manic symptoms may be less likely to recognise or acknowledge any functional impairment. However, these features were present in only a minority of this sample, and those with severe manic or psychotic symptoms would be less able to consent to participate (or participation would be delayed until insight had returned). Lastly, we do not report medications in this paper - this is partly because many young people were drug free at the time of assessment, and those who were prescribed medications had often been on low doses of standard treatments or had not been taking medication for very long; also, non-adherence rates are high in youth (Tacchi and Scott 2006). Taken altogether, this means that impairment due to medication side effects was unlikely to be a significant confounder in this study population, although it is conceivable that lack of or low doses of medication may mean that the level of acute distress at presentation was higher than in more comprehensively medicated samples.

## Conclusions

In youth presenting with a mood disorder for the first time, nearly four out of ten individuals reported high levels of distress or disability, but about 30% of cases were discordant (high distress/low impairment and vice versa). Gender rather than diagnostic sub-type was strongly associated with distress levels, whilst older age showed a significant association with functional impairment. Co-morbid mental disorders were common but were not as important as alcohol and substance use, especially regular cannabis use, as the best predictors of distress and impairment. The major implications of this study are as follows: (a) mood disorders in youth are not mild problems and require active treatment, and (b) the co-occurrence of substance use will complicate both the assessment of the consequences of mood disorder for the individual and the options for intervention.

## Abbreviations

BD: Bipolar disorder
DSM IV: Diagnostic and statistical manual, 4th edition
Kessler-10/K-10: Kessler psychological distress scale
MNLR: Multinomial logistic regression
SD: Standard deviation
UP: Unipolar depression
WSAS: Work and social adjustment scale.
